# Effects of some anti-diabetic and cardioprotective agents on proliferation and apoptosis of human coronary artery endothelial cells

**DOI:** 10.1186/1475-2840-11-27

**Published:** 2012-03-21

**Authors:** Linnéa Eriksson, Özlem Erdogdu, Thomas Nyström, Qimin Zhang, Åke Sjöholm

**Affiliations:** 1 Department of Clinical Science and Education, Södersjukhuset, Karolinska Institutet, Stockholm SE-11883, Sweden

**Keywords:** Diabetes, Endothelium, Coronary artery disease, Insulin, Statin, Metformin, Lipids, AT_1_-receptor antagonist, Peroxisome proliferator-activated receptor gamma agonist

## Abstract

**Background:**

The leading cause of death for patients suffering from diabetes is macrovascular disease. Endothelial dysfunction is often observed in type 2 diabetic patients and it is considered to be an important early event in the pathogenesis of atherogenesis and cardiovascular disease. Many drugs are clinically applied to treat diabetic patients. However, little is known whether these agents directly interfere with endothelial cell proliferation and apoptosis. This study therefore aimed to investigate how anti-diabetic and cardioprotective agents affect human coronary artery endothelial cells (HCAECs).

**Methods:**

The effect of anti-diabetic and cardioprotective agents on HCAEC viability, proliferation and apoptosis was studied. Viability was assessed using Trypan blue exclusion; proliferation in 5 mM and 11 mM of glucose was analyzed using [^3^H]thymidine incorporation. Lipoapoptosis of the cells was investigated by determining caspase-3 activity and the subsequent DNA fragmentation after incubation with the free fatty acid palmitate, mimicking diabetic lipotoxicity.

**Results:**

Our data show that insulin, metformin, BLX-1002, and rosuvastatin improved HCAEC viability and they could also significantly increase cell proliferation in low glucose. The proliferative effect of insulin and BLX-1002 was also evident at 11 mM of glucose. In addition, insulin, metformin, BLX-1002, pioglitazone, and candesartan significantly decreased the caspase-3 activity and the subsequent DNA fragmentation evoked by palmitate, suggesting a protective effect of the drugs against lipoapoptosis.

**Conclusion:**

Our results suggest that the anti-diabetic and cardioprotective agents mentioned above have direct and beneficial effects on endothelial cell viability, regeneration and apoptosis. This may add yet another valuable property to their therapeutic effect, increasing their clinical utility in type 2 diabetic patients in whom endothelial dysfunction is a prominent feature that adversely affect their survival.

## Background

The prevalence of diabetes among adults worldwide was estimated in 2010 to 6.4%, thus affecting 285 million adults. This figure is predicted to rise to 7.7%, in numbers 439 million, by 2030 [1]. In patients with diabetes, the major cause of death is macrovascular disease [2,3], and in individuals with type 2 diabetes, the main etiology for up to 75% of the mortality is atherosclerotic cardiovascular disease [4]. In contrast to microangiopathies (*e.g*. nephropathy and retinopathy), where the causal relation to hyperglycemia is well supported, the link between hyperglycemia and macroangiopathy is uncertain, at least in terms of the possibility of reducing macrovascular morbidity solely by reducing hyperglycemia. Most patients with diabetes are consequently being treated with one or more antidiabetic drugs, a lipid-lowering statin and an ACE inhibitor or angiotensin receptor antagonist for hypertension and/or albuminuria.

The endothelium is composed of a monolayer of cells that line the lumen of blood vessels and form a physical barrier between circulating blood and the vascular smooth muscle cells. The endothelium plays a very important role in maintenance of vascular integrity by protecting the vessels from activation of clotting and proinflammatory factors. It also participates in the regulation of blood flow and blood pressure [5]. Loss of physiological features of the endothelium, such as its preference to support vasodilatation, fibrinolysis and antiaggregation, is referred to as endothelial dysfunction [6], which has been observed in diabetes (type 1 and type 2), in obesity and in patients with insulin resistance. In fact, the extent of endothelium-dependent vasodilatation correlates in obese and insulin resistant subjects with their individual insulin sensitivity [7,8]. Endothelial dysfunction has thus emerged as an important early target for preventing atherosclerosis and cardiovascular disease [9].

Hyperglycemia and hyperlipidemia are important factors in the development of endothelial dysfunction. Free fatty acids (FFAs), formed during lipolysis from triglycerides, and hyperglycemia are known to impair the endothelial-dependent vasodilatation [10,11]. Increased plasma levels of FFAs and glucose are characteristic features in patients with type-2 diabetes. Both factors are known induce apoptosis of endothelial cells and endothelial cell death is believed to be involved in, and contribute to, endothelial dysfunction and atherosclerosis [12-14].

Current anti-diabetes drugs are mainly aimed to correct hyperglycemia by promoting pancreatic β-cell insulin secretion, increasing insulin sensitivity, or reducing intestinal glucose uptake and hepatic gluconeogenesis. Apart from insulin, glimepiride, a third generation sulfonylurea [15], pioglitazone, a peroxisome proliferator-activated receptor gamma (PPAR-γ) agonist, and a member of the thiazolidinedione family (TZD) [16], and metformin, a biguanide, are used for treatment of type 2 diabetes [9]. Candesartan, an angiotensin II receptor antagonist, and rosuvastatin, a competitive inhibitor of the enzyme HMG-CoA reductase, are used for management of hypertension and hyperlipidemia, respectively [17,18]. In addition, we have also studied BLX-1002, a novel thiazolidinedione with no structural resemblance to other TZDs [19], which does not appear to affect PPARs. There is evidence that BLX-1002 can improve hyperglycemia in diabetic animal models without the body weight gain typically associated with PPARγ-mediated adipocyte differentiation [19,20]. Not much is known about BLX-1002, but it has been shown to potentiate insulin secretion from islets in a phosphatidylinositol 3-kinase (PI3K)-dependent manner. BLX-1002 also activates AMP-activated protein kinase (AMPK) [20] perhaps through its ability to inhibit the mitochondrial complex 1 [19,21].

Compared to studies on the actions of the above drugs on glycemia, little has been done regarding their direct actions on proliferation and apoptosis of human coronary artery endothelial cells (HCAECs). Understanding the effects of these agents is important as endothelial growth and apoptosis are involved in endothelial repair and function. Disruption of the intimal layer subjects the arterial wall to greater risk for macrovascular disease [14], the most common etiology for morbidity and mortality in diabetic patients [15]. Therefore, the aim of our study was to investigate the effects of drugs used in treatment of diabetic patients on proliferation and lipotoxicity-induced apoptosis in HCAECs.

## Methods

### Materials

Clonetics™ HCAECs, culture medium EGM-2 MV and cell culture supplements were purchased from Lonza (Basel, Switzerland). Insulin lispro was from Eli Lilly and Company (Indianapolis, IN), C-peptide was generously provided by Prof. John Wahren (Creative Peptides, Inc., Stockholm, Sweden), and pioglitazone was graciously donated by Takeda Pharmaceuticals North America, Inc. (Lincolnshire, IL). Metformin, glimepiride, sodium palmitate, and bovine serum albumin (BSA) (fatty acid free) were purchased from Sigma-Aldrich (St. Louis, MO). Rosuvastatin and candesartan were kindly given by Astra-Zeneca (London, United Kingdom). BLX-1002 was graciously donated by Bexel Pharmaceuticals, Inc. (Union City, CA). [^3^H]thymidine was bought from Amersham Biosciences (Piscataway, NJ), EnzChek^® ^caspase-3 activity assay kits from Molecular Probes^®^, Life Technologies (Carlsbad, CA), DNA fragmentation ELISA kits from Roche Diagnostics (Mannheim, Germany), and DC™ Protein Assay from BioRad Laboratories (Hercules, CA).

### Cell culture

HCAECs, isolated from normal human coronary arteries [22], were grown in EGM-2 MV medium supplemented with hydrocortisone, human epidermal growth factor (hEGF), 5% fetal bovine serum (FBS), vascular endothelial growth factor (VEGF), human fibroblast growth factor (hFGF)-B, R3-insulin-like growth factor (IGF)-1, ascorbic acid and gentamicin/amphotericin-B at 37°C in a humidified (5% CO^2^, 95% air) atmosphere as recommended by the supplier. Passage 5-12 were used in the study for evaluation of cell viability, [^3^H]thymidine incorporation rates, DNA fragmentation ELISA and caspase-3 activity assays. Confluent cultures were detached by trypsination and seeded onto tissue culture dishes and grown until 80-90% confluence. Cells were incubated overnight in serum-deficient EGM medium containing 0.5% FBS and 2 mM L-glutamine prior to 24 or 48 h incubation in the presence or absence of the desired agents. The use of the cells was approved by the Research Ethics Committee, Stockholm South, Dnr: 232/03.

### Cell viability

Cells were incubated with serum deficient medium, in the presence or absence of the drugs for 48 h. Cell number was manually counted in a hemocytometer and cell viability assessed by Trypan blue exclusion.

### [^3^H]thymidine incorporation

Rates of [^3^H]thymidine incorporation into DNA were analyzed as previously described [22,23] and used as a measure of DNA synthesis. In brief, cells were cultured until 80% confluence. After serum starvation over night, cells were incubated in the presence of the agents or vehicle for 24 h at 5 mM or 11 mM glucose to simulate a normoglycemic and hyperglycemic milieu, respectively. Cells were pulsed with [^3^H]thymidine (1 μCi/ml) 8 h prior to the end of the incubation. Cells were then collected and homogenized through ultrasonication. The labeled cells were precipitated in ice-cold 10% trichloroacetic acid (TCA). The precipitate was washed with 10% TCA and [^3^H]thymidine incorporation into DNA was measured using a microplate scintillation and luminescence counter (Wallac MicroBeta^® ^Trilux, PerkinElmer) [24]. The protein concentration of the samples was measured using DC™ Protein Assay (Bio-Rad Laboratories).

### Caspase-3 activity

Cells were cultured to 90% confluence. After incubation in serum deficient medium overnight, cells were pretreated for 1 hour with the drugs or solvents, after which the incubation was continued for 24 h in the presence of 0.125 mM palmitate/0.25% BSA or vehicle [13]. Caspase-3 activity, a measure of apoptosis, was evaluated using the EnzChek^® ^Caspase-3 Assay Kit (Molecular Probes^®^, Life Technologies) according the manufacturer's instructions. The assay is based on the 7-amino-4-methylcoumarin-derived substrate Z-DEVD-AMC, which yields a fluorescent product (excitation/emission ~342/441 nm) upon proteolytic cleavage by active caspase-3. All results were normalized to the protein concentration of the corresponding sample using DC™ Protein Assay (Bio-Rad Laboratories).

### DNA fragmentation

Cells were cultured to 90% confluence and treated in the same way as described for the caspase-3 activity assay. Cell apoptosis was analyzed using the Cell Death Detection Kit plus (Roche Diagnostics) according the manufacturer's instructions. The Cell Death Detection Kit measures cytoplasmic DNA-histone nucleosome complexes generated during apoptotic DNA fragmentation. Samples were measured at 405 nm and corrected for background signals caused by irregular microtiter plates or light scattering due to solid particles in the solution at the reference wavelength of 492 nm. Separate wells were seeded for protein concentration measurements using DC™ Protein Assay (Bio-Rad Laboratories). Absorbance data was normalized to the protein concentration of the corresponding treatment.

### Statistical analysis

Results are expressed as mean ± SEM. Statistical analysis was performed using Student's *t*-test or ANOVA, as appropriate. P < 0.05 was considered statistically significant.

## Results

Agents that were used in this study were the following: insulin lispro (100 pM, 1 nM), C-peptide (1 nM), pioglitazone (2.5 μM), metformin (500 μM), glimepiride (1 μM), rosuvastatin (10 nM), candesartan (100 nM) and BLX-1002 (1 μM). Of the compounds tested, we were unable to detect any effect of C-peptide on either proliferation or apoptosis of the cells. All agents have been tested in all conditions, but only compounds that were able to exert significant effects are displayed in the graphs.

### Cell viability of HCAECs is increased by the agents

**Figure 1:**
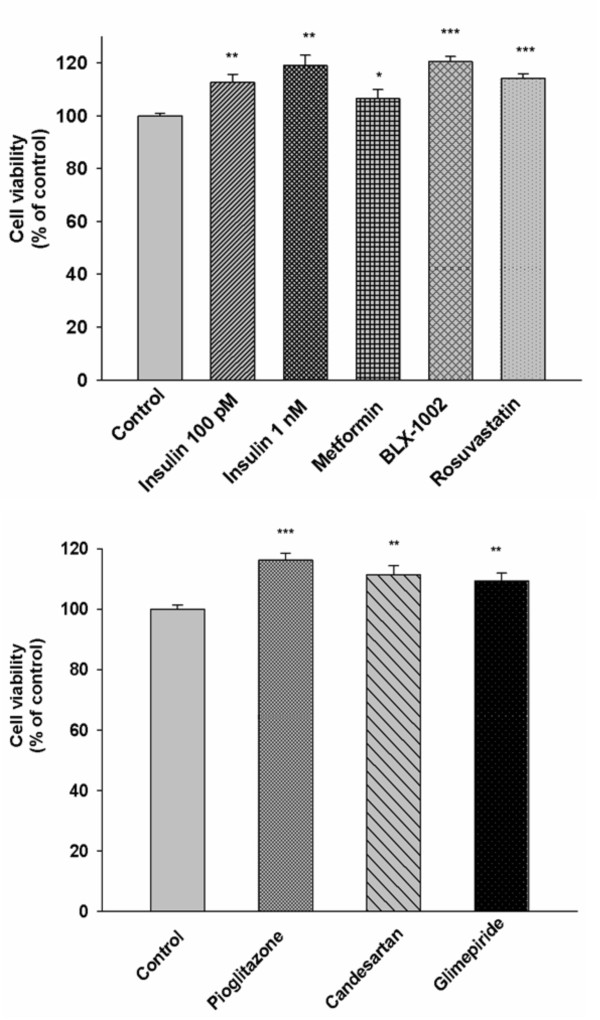
**Insulin, metformin, BLX-1002, rosuvastatin, pioglitazone, candesartan and glimepiride increase HCAEC viability**. Cells were incubated for 48 h in medium supplemented with 0.5% FBS in the presence or absence of insulin lispro (100 pM and 1 nM), metformin (500 μM), BLX-1002 (1 μM), rosuvastatin (10 nM), pioglitazone (2.5 μM), candesartan (100 nM) and glimepiride (1 μM). Viability was assessed with Trypan blue exclusion. * denotes P < 0.05, ** denotes P < 0.01, *** denotes P < 0.001 for chance differences compared to controls by Student's *t*-test.

As shown in Figure 1, among the agents tested, BLX-1002 and insulin (the latter at both 100 pM and 1 nM) increased cell viability. Exposure of the cells to metformin also resulted in a slight, but statistically significant, increase in cell viability. Under the same conditions, cell viability was also augmented by rosuvastatin, pioglitazone, candesartan and glimepiride.

### Insulin, BLX-1002, metformin and rosuvastatin stimulate proliferation of HCAECs at normal glucose concentration

**Figure 2:**
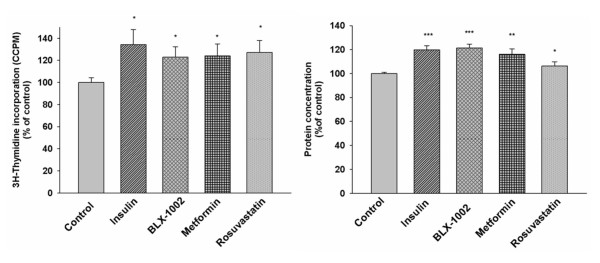
**Insulin, BLX-1002, metformin and rosuvastatin stimulate HCAEC proliferation at normal glucose concentration**. In order to study the effects of agents on proliferation of HCAECs, cells were incubated at 5 mM of glucose in medium supplemented with 0.5% FBS for 24 h in the presence or absence of insulin lispro (100 pM), metformin (500 μM), BLX-1002 (1 μM), rosuvastatin (10 nM). Eight hours prior to the end of the incubation, cells were pulsed with [^3^H]thymidine. Cells were then harvested and [^3^H]thymidine incorporation into DNA was measured using a microplate scintillation & luminescence counter, and the protein concentration of the samples was measured using DC™ Protein Assay. * denotes P < 0.05, ** denotes P < 0.01, *** denotes P < 0.001 for chance differences vs. controls by Student's *t*-test.

Since the increased viability noted above (Figure 1) could be explained by increased proliferation, decreased apoptosis, or a combination thereof, we investigated if the drugs exert any effect on HCAECs' DNA synthesis. Rates of [^3^H]thymidine incorporation were analyzed after a 24 hour exposure to the agents at 5 mM glucose. The mitogenic effect of the compounds was also verified by measuring protein concentrations of the samples to reflect an increase in cell number. As shown in Figure 2, insulin, BLX-1002, metformin and rosuvastatin increased both DNA synthesis and protein concentration significantly.

### Insulin and BLX-1002 stimulate proliferation of HCAECs at high glucose

**Figure 3:**
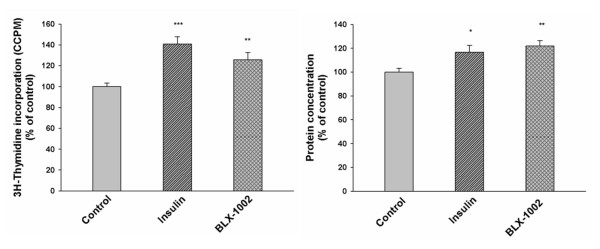
**Insulin and BLX-1002 stimulate HCAEC proliferation in high glucose**. HCAECs were incubated at 11 mM of glucose in medium supplemented with 0.5% FBS for 24 h in the presence or absence of insulin lispro (100 pM) or BLX-1002 (1 μM). Eight hours prior to the end of the incubation, cells were pulsed with [^3^H]thymidine. Cells were then harvested and [^3^H]thymidine incorporation into DNA was measured using a microplate scintillation & luminescence counter and the protein concentration of the samples was measured using DC™ Protein Assay. * denotes P < 0.05, ** denotes P < 0.01, *** denotes P < 0.001 for a chance difference vs. control by Student's *t*-test.

Since insulin, BLX-1002, metformin and rosuvastatin were able to increase the proliferation of HCAECs in 5 mM glucose (Figure 2), we further investigated whether they had any effect on cell proliferation at high glucose. HCAECs were therefore exposed to 11 mM of glucose, to simulate a diabetic setting, in the presence or absence of the agents. Similar to the effect observed at 5 mM glucose, insulin and BLX-1002 were both able to increase DNA synthesis and protein concentration (Figure 3). In contrast to their effects at 5 mM glucose, metformin and rosuvastatin did not exert any effect on cell proliferation under these conditions (data not shown).

### Insulin, BLX-1002, metformin, pioglitazone and candesartan suppress lipoapoptosis in HCAECs

**Figure 4:**
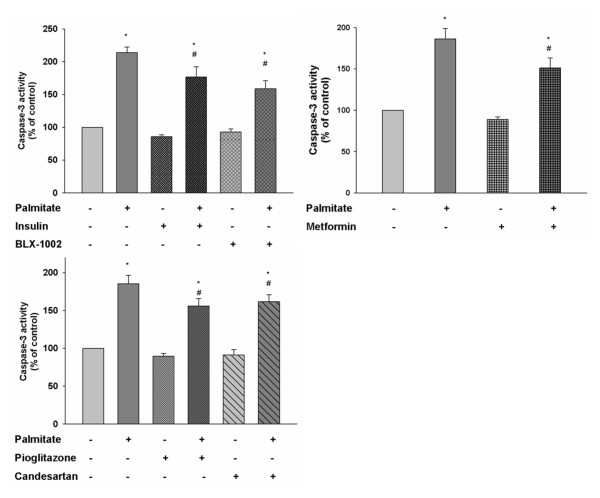
**Insulin, metformin, BLX-1002, pioglitazone and candesartan protect HCAECs against palmitate-induced caspase-3 activation**. Palmitate-induced caspase-3 activity, a measure of apoptosis, was evaluated using the EnzChek^® ^Caspase-3 Assay Kit. HCAECs were incubated in medium containing 5 mM glucose, supplemented with 0.5% FBS, with or without insulin lispro (1 nM), BLX-1002 (1 μM), metformin (500 μM), pioglitazone (2.5 μM), candesartan (100 nM) in the presence of 0.125 mM palmitate or vehicle (ethanol and BSA) for 24 h. * denotes P < 0.05 for a chance difference vs. controls by ANOVA. # denotes P < 0.05 for a chance difference vs. palmitate by ANOVA.

Plasma FFA levels are increased in type-2 diabetic patients and the FFA palmitate is known to induce apoptosis of endothelial cells [13]. We therefore evaluated how the agents affected the activity of cleaved caspase-3, a crucial mediator of apoptosis [25], in HCAECs after 24 hour exposure to 0.125 mM palmitate. As shown in Figure 4, palmitate significantly increased caspase-3 activity, thus confirming its pro-apoptotic effect, in HCAECs. Insulin, BLX-1002, metformin, pioglitazone and candesartan significantly decreased the palmitate-induced caspase-3 activation, suggesting a protective effect of the drugs against lipotoxicity in HCAECs. Glimepiride significantly decreased basal caspase-3 activity with ~20% (data not shown), which might explain why an increased viability of the cells in the presence of glimepiride is noted (Figure 1).

**Figure 5:**
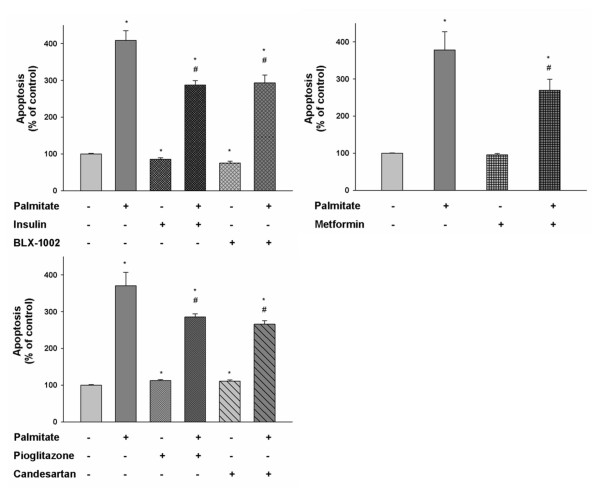
**Insulin, metformin, BLX-1002, pioglitazone and candesartan protect HCAECs against palmitate-induced DNA fragmentation**. To further corroborate the protective effects of the agents against lipotoxicity-induced apoptosis, DNA fragmentation was evaluated using the Cell Death Detection kit ELISA^plus^. Cells were exposed for 24 h to 0.125 mM palmitate or vehicle at 5 mM glucose with or without insulin lispro (1 nM), BLX-1002 (1 μM), metformin (500 μM), pioglitazone (2.5 μM), candesartan (100 nM). * denotes P < 0.05 for a chance difference vs controls by ANOVA, # denotes P < 0.05 for a chance difference vs palmitate by ANOVA.

To corroborate the anti-apoptotic effect of the agents above, we investigated their influence on palmitate-induced DNA fragmentation by analyzing cytoplasmic DNA-histone nucleosome complexes generated during apoptosis. Palmitate alone gave rise to a markedly increased apoptosis of the HCAECs (Figure 5). Co-incubation with insulin, BLX-1002, metformin, pioglitazone or candesartan significantly countered the palmitate-induced apoptosis, thus confirming the protective effect of these agents against lipoapoptosis in HCAECs.

## Discussion

Our major findings in this study are that insulin, BLX-1002, metformin and rosuvastatin exert positive effects on regeneration and viability of HCAECs. Insulin, BLX-1002, metformin, pioglitazone and candesartan also conferred protection of HCAECs against lipotoxicity.

### Proliferation of HCAECs is induced in normal and high glucose levels

By studying how the agents affect DNA synthesis, through analysis of the incorporation of radiolabeled thymidine into DNA, we found that insulin, BLX-1002, metformin and rosuvastatin increased the HCAECs' DNA synthesis and subsequently the protein concentration in 5 mM glucose. Taken together, this suggests that the above agents stimulate HCAEC proliferation at normal glucose concentrations. The mitogenic effect seen with the compounds probably explain, at least in part, the increased viability.

Kageyama *et al. *recently showed that when HCAECs were exposed to glucose concentrations ranging from 5.6-16.7 mM apoptosis was dose dependently increased. This was also coupled to an increased expression of the death receptors TNF-R1 and Fas [26]. High glucose is also known to cause oxidative stress through generation of reactive oxygen species (ROS) in HCAECs as recently shown by Serizawa *et al. *[27]. This might be due to on uncoupling of endothelial nitric oxide synthase (eNOS) and increased activity of the NADPH oxidase [27]. Increased levels of glucose might also cause proliferative dysfunction of endothelial cells [28,29], which is believed to contribute to premature development of atherosclerosis [30]. We therefore wanted to test if the above agents also stimulate HCAEC proliferation at 11 mM glucose, simulating a diabetic milieu. We found that insulin and BLX-1002 were the only two agents that could promote cell proliferation in high glucose. The mitogenic effect of the agents in HCAECs may prove beneficial in dampening or delaying coronary atherosclerosis, by rapidly covering a vascular wall lesion with endothelium and thus protecting it from further atherothrombotic events. It may also be favorable for diabetic patients with coronary artery disease undergoing percutaneous coronary revascularization and treated with drug-eluting stents. It is believed that delayed re-endothelization is a major underlying problem for late-stent thrombosis [31] and that rapid re-endothelization is essential for preventing restenosis, which is the major limitation with this treatment [32].

The underlying mechanisms involved in the agents' proliferative effects were not investigated in this scanning study, but will be subject to future work. However, the effects are consistent with previous findings insofar that insulin at supraphysiological concentrations increases [^3^H]thymidine incorporation in endothelial cells [33]. Insulin and rosuvastatin have also been shown to activate the PI3K/Akt pathway, a pathway that is well known to be involved in cell survival and proliferation [18,22,34-36]. BLX-1002 is a novel compound of the TZD family, but with no apparent PPAR affinity. We have previously demonstrated that it can signal through PI3K and is able to activate AMPK in insulin-secreting cells [20]. Also, metformin is a known activator of AMPK, and AMPK activates eNOS [37], an important factor for proliferation of HCAECs [22].

### HCAECs are protected from lipoapotosis

Plasma levels of FFAs are typically elevated in diabetic patients and FFAs are known to impair endothelial-dependent vasodilatation [11,38]. FFAs are also known to cause apoptosis of endothelial cells [13,38]. Apoptosis of HCAECs might disrupt the endothelial monolayer of the coronary artery wall and might contribute to a proinflammatory environment that could lead to premature endothelial dysfunction and unfolding of coronary atherosclerosis [13,38]. We therefore addressed whether the above agents protect HCAECs against apoptotic cell death caused by the fatty acid palmitate, simulating diabetic hyperlipidemia. Our results are commensurate with other reports in the literature. Insulin has previously been shown to protect human umbilical vein endothelial cells (HUVECs) from palmitate-induced apoptosis through activation of the PI3K/Akt pathway [38]. BLX-1002, whose functions are essentially unknown, has been shown to promote insulin secretion in pancreatic β-cells, an effect that involves PI3K and AMPK activation [20]. Since both PI3K and AMPK convey anti-apoptotic signals in other cell types [38-41], these pathways may also be operative in HCAECs. This also holds true for metformin, which can activate AMPK and indeed, specific activation of AMPK has been shown to protect bovine aortic endothelial cells from palmitate-induced apoptosis [41]. The anti-apoptotic effect of pioglitazone observed in the present study is consistent with findings in both HUVECs and endothelial progenitor cells (EPCs) [42,43]. Although the mechanisms behind such effect remain unclear and will be addressed in forthcoming studies, palmitate induces ROS in endothelial cells [41] and pioglitazone has been shown to decrease ROS formation in such cells [44]. This might contribute to its anti-apoptotic effect since oxidative stress is known to induce cell death [14,44]. Pioglitazone has also been shown to protect HCAECs from tumor necrosis factor alpha induced caspase-3 activity and apoptosis [45]. To the best of our knowledge, there is no literature regarding putative effects of candesartan on lipoapoptosis of endothelial cells. But, in support to our findings, candesartan has been shown to protect from hypoxia-induced endothelial cell apoptosis through pathways involving increased eNOS expression and decreased caspase-3 activity [17]. Candesartan's ability to modulate the caspase-3 activity might also contribute to its positive effects on cell viability.

## Conclusions

Abnormal proliferation and apoptosis of endothelial cells are involved in endothelial dysfunction, damage and repair. It thus contributes to the premature development of atherosclerosis and vascular complications in diabetes, making it important to understand the influence of currently applied anti-diabetic and cardioprotective agents on endothelial function. Our results suggest that drugs such as insulin, metformin, rosuvastatin, pioglitazone and candesartan that are oftentimes used in the clinical management of diabetic patients have direct and beneficial effects human coronary artery endothelial cells by promoting their proliferation and conferring protection against lipotoxicity. These findings may add yet another valuable property to their therapeutic effects, increasing their clinical utility in type 2 diabetic patients in whom endothelial dysfunction is a prominent feature that adversely affect their survival.

## Abbreviations

AMPK: AMP-activated protein kinase; BSA: Bovine serum albumin; eNOS: Endothelial NOS; EPC: Endothelial progenitor cells; FBS: Fetal bovine serum; FFA: Free fatty acid; HCAEC: Human coronary artery endothelial cells; HUVEC: Human umbilical vein endothelial cells; hEGF: Human epidermal growth factor; hFGF-B: Human fibroblast growth factor-B; IGF-1: R3-insulin-like growth factor-1; NO: Nitric oxide; PI3K: Phosphatidylinositol 3-kinase; PPAR-γ: Peroxisome proliferator-activated receptor gamma; ROS: Reactive oxygen species; TCA: Trichloroacetic acid; TZD: Thiazolidinedione; VEGF: Vascular endothelial growth factor.

## Competing interests

Dr. Sjöholm has received research grants, and provided lectures, consultancies and expert testimony to several pharmaceutical companies involved in diabetes care, such as GlaxoSmithKline, Takeda Pharmaceuticals North America, Schering-Plough, Novo-Nordisk, Eli Lilly, Novartis, Aventis, Sanofi-Synthelabo, Bristol-Myers, Squibb, Merck Sharp & Dohme, Pfizer, Boehringer-Ingelheim, Selena-Fournier, Roche Diagnostics, Astra-Zeneca, Bayer, Pharmacia, and Hässle Pharmaceuticals.

Dr. Sjöholm is also on the National and/or European Advisory Boards in Diabetes Care for Eli Lilly, Takeda Pharmaceuticals, Novartis, Sanofi-Aventis, Merck Sharp & Dohme, Boehringer-Ingelheim and Astra-Zeneca.

## Authors' contributions

LE and ÖE performed the experimental procedures, performed the statistical calculations, and contributed to results interpretation and discussion. TN, QZ and ÅS provided expertise in diabetes and endothelial dysfunction, the *in vitro *model, and conceived, designed and co-ordinated the research plan, respectively. LE, QZ and ÅS wrote the manuscript. LE, TN, QZ and ÅS contributed to discussion and edited the paper prior to submission. All authors read and approved the final manuscript.

## References

[B1] Shaw JE Sicree RA Zimmet PZ Global estimates of the prevalence of diabetes for 2010 and 2030 Diabetes Res Clin Pract 2010 87 1 4 14 10.1016/j.diabres.2009.10.007 19896746

[B2] Sjoholm A Nystrom T Inflammation and the etiology of type 2 diabetes Diabetes Metab Res Rev 2006 22 1 4 10 10.1002/dmrr.568 15991254

[B3] Sjoholm A Nystrom T Endothelial inflammation in insulin resistance Lancet 2005 365 9459 610 612 15708106 10.1016/S0140-6736(05)17912-4

[B4] Dandona P Ghanim H Chaudhuri A Mohanty P Thiazolidinediones-improving endothelial function and potential long-term benefits on cardiovascular disease in subjects with type 2 diabetes J Diabetes Complications 2008 22 1 62 75 10.1016/j.jdiacomp.2006.10.009 18191079

[B5] Calles-Escandon J Cipolla M Diabetes and endothelial dysfunction: a clinical perspective Endocr Rev 2001 22 1 36 52 10.1210/er.22.1.36 11159815

[B6] Avogaro A de Kreutzenberg SV Fadini G Endothelial dysfunction: causes and consequences in patients with diabetes mellitus Diabetes Res Clin Pract 2008 82 Suppl 2 S94 S101 18954919 10.1016/j.diabres.2008.09.021

[B7] Rask-Madsen C King GL Mechanisms of Disease: endothelial dysfunction in insulin resistance and diabetes Nat Clin Pract Endocrinol Metab 2007 3 1 46 56 10.1038/ncpendmet0366 17179929

[B8] Steinberg HO Chaker H Leaming R Johnson A Brechtel G Baron AD Obesity/insulin resistance is associated with endothelial dysfunction. Implications for the syndrome of insulin resistance J Clin Invest 1996 97 11 2601 2610 10.1172/JCI118709 8647954 PMC507347

[B9] Nathanson D Nystrom T Hypoglycemic pharmacological treatment of type 2 diabetes: targeting the endothelium Mol Cell Endocrinol 2009 297 1-2 112 126 10.1016/j.mce.2008.11.016 19038307

[B10] Beckman JA Goldfine AB Gordon MB Garrett LA Creager MA Inhibition of protein kinase Cbeta prevents impaired endothelium-dependent vasodilation caused by hyperglycemia in humans Circ Res 2002 90 1 107 111 10.1161/hh0102.102359 11786526

[B11] Lundman P Tornvall P Nilsson L Pernow J A triglyceride-rich fat emulsion and free fatty acids but not very low density lipoproteins impair endothelium-dependent vasorelaxation Atherosclerosis 2001 159 1 35 41 10.1016/S0021-9150(01)00478-6 11689204

[B12] Ho FM Liu SH Liau CS Huang PJ Lin-Shiau SY High glucose-induced apoptosis in human endothelial cells is mediated by sequential activations of c-Jun NH(2)-terminal kinase and caspase-3 Circulation 2000 101 22 2618 2624 10840014 10.1161/01.cir.101.22.2618

[B13] Chai W Liu Z p38 mitogen-activated protein kinase mediates palmitate-induced apoptosis but not inhibitor of nuclear factor-kappaB degradation in human coronary artery endothelial cells Endocrinology 2007 148 4 1622 1628 17234706 10.1210/en.2006-1068

[B14] Cifarelli V Geng X Styche A Lakomy R Trucco M Luppi P C-peptide reduces high-glucose-induced apoptosis of endothelial cells and decreases NAD(P)H-oxidase reactive oxygen species generation in human aortic endothelial cells Diabetologia 2011 54 10 2702 2712 10.1007/s00125-011-2251-0 21773684

[B15] Hamaguchi T Hirose T Asakawa H Itoh Y Kamado K Tokunaga K Tomita K Masuda H Watanabe N Namba M Efficacy of glimepiride in type 2 diabetic patients treated with glibenclamide Diabetes Res Clin Pract 2004 66 Suppl 1 S129 S132 15563963 10.1016/j.diabres.2003.12.012

[B16] Smith U Pioglitazone: mechanism of action Int J Clin Pract Suppl 2001 121 13 18 11594239

[B17] Matsumoto N Manabe H Ochiai J Fujita N Takagi T Uemura M Naito Y Yoshida N Oka S Yoshikawa T An AT1-receptor antagonist and an angiotensin-converting enzyme inhibitor protect against hypoxia-induced apoptosis in human aortic endothelial cells through upregulation of endothelial cell nitric oxide synthase activity Shock 2003 19 6 547 552 10.1097/01.shk.0000070734.34700.80 12785010

[B18] Li X Yang G Zhao G Wu B Edin ML Zeldin DC Wang DW Rosuvastatin attenuates the elevation in blood pressure induced by overexpression of human C-reactive protein Hypertens Res 2011 34 7 869 875 10.1038/hr.2011.44 21562509 PMC4042242

[B19] Brunmair B Staniek K Lehner Z Dey D Bolten CW Stadlbauer K Luger A Furnsinn C Lipophilicity as a determinant of thiazolidinedione action in vitro: findings from BLX-1002, a novel compound without affinity to PPARs Am J Physiol Cell Physiol 2011 300 6 C1386 C1392 10.1152/ajpcell.00401.2010 21346152

[B20] Zhang F Dey D Branstrom R Forsberg L Lu M Zhang Q Sjoholm A BLX-1002, a novel thiazolidinedione with no PPAR affinity, stimulates AMP-activated protein kinase activity, raises cytosolic Ca2+, and enhances glucose-stimulated insulin secretion in a PI3K-dependent manner Am J Physiol Cell Physiol 2009 296 2 C346 C354 19052259 10.1152/ajpcell.00444.2008

[B21] Brunmair B Staniek K Gras F Scharf N Althaym A Clara R Roden M Gnaiger E Nohl H Waldhausl W Thiazolidinediones, like metformin, inhibit respiratory complex I: a common mechanism contributing to their antidiabetic actions? Diabetes 2004 53 4 1052 1059 10.2337/diabetes.53.4.1052 15047621

[B22] Erdogdu O Nathanson D Sjoholm A Nystrom T Zhang Q Exendin-4 stimulates proliferation of human coronary artery endothelial cells through eNOS-, PKA- and PI3K/Akt-dependent pathways and requires GLP-1 receptor Mol Cell Endocrinol 2009 325 1-2 26 35 20452396 10.1016/j.mce.2010.04.022

[B23] Edwards SJ Reader KL Lun S Western A Lawrence S McNatty KP Juengel JL The cooperative effect of growth and differentiation factor-9 and bone morphogenetic protein (BMP)-15 on granulosa cell function is modulated primarily through BMP receptor II Endocrinology 2008 149 3 1026 1030 18063682 10.1210/en.2007-1328

[B24] Sjoholm A Zhang Q Welsh N Hansson A Larsson O Tally M Berggren PO Rapid Ca2+ influx and diacylglycerol synthesis in growth hormone-mediated islet beta -cell mitogenesis J Biol Chem 2000 275 28 21033 21040 10.1074/jbc.M001212200 10748000

[B25] Nicholson DW Ali A Thornberry NA Vaillancourt JP Ding CK Gallant M Gareau Y Griffin PR Labelle M Lazebnik YA Identification and inhibition of the ICE/CED-3 protease necessary for mammalian apoptosis Nature 1995 376 6535 37 43 10.1038/376037a0 7596430

[B26] Kageyama S Yokoo H Tomita K Kageyama-Yahara N Uchimido R Matsuda N Yamamoto S Hattori Y High glucose-induced apoptosis in human coronary artery endothelial cells involves up-regulation of death receptors Cardiovasc Diabetol 2011 10 73 10.1186/1475-2840-10-73 21816064 PMC3161855

[B27] Serizawa K Yogo K Aizawa K Tashiro Y Ishizuka N Nicorandil prevents endothelial dysfunction due to antioxidative effects via normalisation of NADPH oxidase and nitric oxide synthase in streptozotocin diabetic rats Cardiovasc Diabetol 2011 10 105 10.1186/1475-2840-10-105 22107602 PMC3248842

[B28] Varma S Lal BK Zheng R Breslin JW Saito S Pappas PJ Hobson RW Duran WN Hyperglycemia alters PI3k and Akt signaling and leads to endothelial cell proliferative dysfunction Am J Physiol Heart Circ Physiol 2005 289 4 H1744 H1751 10.1152/ajpheart.01088.2004 15964918 PMC1618822

[B29] McGinn S Saad S Poronnik P Pollock CA High glucose-mediated effects on endothelial cell proliferation occur via p38 MAP kinase Am J Physiol Endocrinol Metab 2003 285 4 E708 E717 12783777 10.1152/ajpendo.00572.2002

[B30] Berk BC Abe JI Min W Surapisitchat J Yan C Endothelial atheroprotective and anti-inflammatory mechanisms Ann N Y Acad Sci 2001 947 93 109 discussion 109-111 11795313 10.1111/j.1749-6632.2001.tb03932.x

[B31] Finn AV Joner M Nakazawa G Kolodgie F Newell J John MC Gold HK Virmani R Pathological correlates of late drug-eluting stent thrombosis: strut coverage as a marker of endothelialization Circulation 2007 115 18 2435 2441 10.1161/CIRCULATIONAHA.107.693739 17438147

[B32] Nickson CM Doherty PJ Williams RL Novel polymeric coatings with the potential to control in-stent restenosis-an in vitro study J Biomater Appl 2010 24 5 437 452 10.1177/0885328208099338 19033327

[B33] Gousseva N Kugathasan K Chesterman CN Khachigian LM Early growth response factor-1 mediates insulin-inducible vascular endothelial cell proliferation and regrowth after injury J Cell Biochem 2001 81 3 523 534 10.1002/1097-4644(20010601)81:3<523::AID-JCB1066>3.0.CO;2-E 11255235

[B34] Enomoto S Sata M Fukuda D Nakamura K Nagai R Rosuvastatin prevents endothelial cell death and reduces atherosclerotic lesion formation in ApoE-deficient mice Biomed Pharmacother 2009 63 1 19 26 10.1016/j.biopha.2007.11.002 18162361

[B35] Engelman JA Luo J Cantley LC The evolution of phosphatidylinositol 3-kinases as regulators of growth and metabolism Nat Rev Genet 2006 7 8 606 619 10.1038/nrg1879 16847462

[B36] Zeng G Nystrom FH Ravichandran LV Cong LN Kirby M Mostowski H Quon MJ Roles for insulin receptor, PI3-kinase, and Akt in insulin-signaling pathways related to production of nitric oxide in human vascular endothelial cells Circulation 2000 101 13 1539 1545 10747347 10.1161/01.cir.101.13.1539

[B37] Davis BJ Xie Z Viollet B Zou MH Activation of the AMP-activated kinase by antidiabetes drug metformin stimulates nitric oxide synthesis in vivo by promoting the association of heat shock protein 90 and endothelial nitric oxide synthase Diabetes 2006 55 2 496 505 10.2337/diabetes.55.02.06.db05-1064 16443786

[B38] Piro S Spampinato D Spadaro L Oliveri CE Purrello F Rabuazzo AM Direct apoptotic effects of free fatty acids on human endothelial cells Nutr Metab Cardiovasc Dis 2008 18 2 96 104 10.1016/j.numecd.2007.01.009 17560770

[B39] Hermann C Assmus B Urbich C Zeiher AM Dimmeler S Insulin-mediated stimulation of protein kinase Akt: a potent survival signaling cascade for endothelial cells Arterioscler Thromb Vasc Biol 2000 20 2 402 409 10.1161/01.ATV.20.2.402 10669636

[B40] Franke TF Kaplan DR Cantley LC PI3K: downstream AKTion blocks apoptosis Cell 1997 88 4 435 437 10.1016/S0092-8674(00)81883-8 9038334

[B41] Kim JE Kim YW Lee IK Kim JY Kang YJ Park SY AMP-activated protein kinase activation by 5-aminoimidazole-4-carboxamide-1-beta-D-ribofuranoside (AICAR) inhibits palmitate-induced endothelial cell apoptosis through reactive oxygen species suppression J Pharmacol Sci 2008 106 3 394 403 10.1254/jphs.FP0071857 18360094

[B42] Artwohl M Holzenbein T Furnsinn C Freudenthaler A Huttary N Waldhausl WK Baumgartner-Parzer SM Thiazolidinediones inhibit apoptosis and heat shock protein 60 expression in human vascular endothelial cells Thromb Haemost 2005 93 5 810 815 15886792 10.1160/TH04-09-0615

[B43] Gensch C Clever YP Werner C Hanhoun M Bohm M Laufs U The PPAR-gamma agonist pioglitazone increases neoangiogenesis and prevents apoptosis of endothelial progenitor cells Atherosclerosis 2007 192 1 67 74 10.1016/j.atherosclerosis.2006.06.026 16876172

[B44] Fujisawa K Nishikawa T Kukidome D Imoto K Yamashiro T Motoshima H Matsumura T Araki E TZDs reduce mitochondrial ROS production and enhance mitochondrial biogenesis Biochem Biophys Res Commun 2009 379 1 43 48 10.1016/j.bbrc.2008.11.141 19084501

[B45] Chen J Li D Zhang X Mehta JL Tumor necrosis factor-alpha-induced apoptosis of human coronary artery endothelial cells: modulation by the peroxisome proliferator-activated receptor-gamma ligand pioglitazone J Cardiovasc Pharmacol Ther 2004 9 1 35 41 10.1177/107424840400900i106 15094967

